# Evaluation of pulmonary perfusion by SPECT imaging using an endothelial cell tracer in supine humans and dogs

**DOI:** 10.1186/s13550-016-0198-3

**Published:** 2016-05-27

**Authors:** Xavier Levac, François Harel, Vincent Finnerty, Quang T. Nguyen, Myriam Letourneau, Sophie Marcil, Alain Fournier, Jocelyn Dupuis

**Affiliations:** Research Center, Montreal Heart Institute, 5000, Belanger, Montreal, QC H1T 1C8 Canada; Department of Medicine, Université de Montréal, Montréal, Québec Canada; Department of Nuclear Medicine, Université de Montréal, Montréal, Québec Canada; INRS-Institut Armand Frappier, Laval, Québec Canada

**Keywords:** Pulmonary vascular endothelium, Molecular imaging, Nuclear medicine, Adrenomedullin, Lung perfusion

## Abstract

**Background:**

Pulmonary perfusion is not spatially homogeneously distributed, and its variations could be of diagnostic value in lung vascular disease. PulmoBind is a ligand of the adrenomedullin receptor densely expressed in endothelial cells of lung capillaries. The aim of this study was to evaluate spatial distribution of human lung perfusion by using this novel molecular tracer of the pulmonary vascular endothelium.

**Methods:**

Normal humans (*n* = 19) enrolled into the PulmoBind phase I trial were studied (Clinicaltrials.gov.NCT01539889). They were injected with ^99m^Tc-PulmoBind for SPECT imaging. Results were compared with ^99m^Tc-PulmoBind in quadruped mammals (dogs, *n* = 5). Imaging was performed in the supine position and distribution of activity was determined as a function of cumulative voxels along the different anatomical planes.

**Results:**

PulmoBind uptake in humans was 58 ± 1 % (mean ± SEM) of the injected dose. Dorsal activity was 18.1 ± 2.1 % greater than ventral, and caudal activity was 25.7 ± 1.6 % greater than cranial. Lateral activity was only mildly higher than medial by 7.0 ± 1.0 %. In supine dogs, similar but higher PulmoBind gradients were present: dorsal 28.6 ± 2.5 %, caudal 34.1 ± 5.0 % and lateral 18.1 ± 2.0 %.

**Conclusions:**

The perfused pulmonary circulation of supine humans, assessed by an adrenomedullin receptor ligand, is not homogeneously distributed with more prominent distribution in dorsal and caudal regions. It is qualitatively similar to a supine quadruped mammal confirming the presence of a microcirculatory gravitational perfusion gradient detectable with this tracer. Future studies are needed to determine if this novel endothelial cell tracer could be used to detect physiologic and pathologic variations of lung perfusion such as in pulmonary hypertension.

**Clinical trial:**

ClinicalTrial.gov, NCT01539889

## Background

It is well established that pulmonary perfusion is not homogeneously distributed [1, 2]. Both gravity-dependent and gravity-independent factors contribute to the spatial distribution of pulmonary perfusion, their relative importance being the subject of passionate scientific debates [3–7]. There is however consensus that this heterogeneity confers great capacitance to the pulmonary vasculature and the possibility to increase (recruit) tissue perfusion for gas and metabolic exchanges in response to increasing cardiac output. A study of the spatial distribution of pulmonary perfusion may be of great value in evaluating the pulmonary vascular reserve and its variations in health and disease.

Various imaging modalities have been utilized to study the spatial distribution of pulmonary perfusion in humans including computerized tomography (CT), magnetic resonance imaging (MRI), positron emission tomography and single-photon emission computed tomography (SPECT). All of these methods have relied on the use of physical and structural agents distributed within the pulmonary circulation and whose external signals are reflections of flow. There is therefore currently no clinically validated imaging agent that relies on biologic properties of the endothelium and can quantitatively assess the spatial distribution of perfusion to the metabolically active pulmonary circulation and its variations in health and disease.

We developed a new tracer that can provide molecular SPECT imaging of the perfused lung capillaries by binding to the pulmonary vascular endothelium. This tracer is an adrenomedullin receptor ligand called PulmoBind. PulmoBind was specifically developed for molecular SPECT imaging of the pulmonary circulation by labelling with ^99m^Tc [8]. Adrenomedullin receptors are abundantly distributed in human alveolar capillaries mostly at the luminal surface of the vascular endothelium [9–11] and are responsible for important lung clearance of this peptide [12]. A safety and dosimetric study of PulmoBind in human subjects was recently completed [13]. PulmoBind was found to be safe with rapid and important lung uptake. The majority of injected PulmoBind (~58 %) is retained by the human lung.

The primary aim of this study was to analyse the spatial distribution of the perfused lung capillaries in healthy human subjects by molecular SPECT imaging using PulmoBind. A secondary aim was to compare this distribution to that obtained with SPECT PulmoBind imaging in a quadruped mammal (dogs). Evaluation of the geometric distribution of lung perfusion could be useful in the earlier diagnosis of lung vascular disorders such as pulmonary hypertension. Indeed, subjects suffering from pulmonary hypertension are diagnosed on average 2 years after their initial medical contact.

## Methods

We studied subjects enrolled into the PulmoBind phase I safety study (study number: PB-01) conducted at the Montreal Heart Institute and registered at Clinicaltrials.gov (NCT01539889) [13]. Twenty healthy non-smoking volunteers (18 men, 2 women) with a mean age of 34 ± 15 years (mean ± SD) and weight of 75 ± 14 kg (mean ± SD) were included into study PB-01. In one male subject, SPECT imaging was not analysable due to improper injection of the tracer so that 19 subjects were included in the present study. They had normal biochemistry and haematology, echocardiogram, chest X-rays and respiratory function tests. The study was approved by the institutional ethic and scientific review boards and was done in accordance with the Declaration of Helsinki and International Conference on Harmonization/Good Clinical Practice guidelines. Written informed consent was obtained from each participant.

Details concerning the synthesis and radiolabelling of PulmoBind were previously described thoroughly [8]. The subjects received between 5 and 15 mCi of ^99m^Tc-PulmoBind with a mean radiochemical purity of 95 ± 4 % (mean ± SD). Prior to administration, total injection syringe activity was determined. The participants were lying in supine position 30 min before administration. ^99m^Tc-PulmoBind was injected intravenously over 3 min with an automatic injector. Ninety minutes later, SPECT imaging was performed with an E-cam Dual Head Gamma Camera equipped with a low-energy and high-resolution collimator. Acquisition parameters were 128 × 128 pixels matrix, 64 frames over 360°, zoom 1.0, pixels size of 2.67 mm, 35 s per acquisition view and total imaging time of 20 min. Following the imaging protocol, residual activity of the syringe was measured to determine the injected activity.

### Image analysis

Tomographic datasets were reconstructed using 3D ordered subset expectation maximization (OSEM) iterative reconstruction algorithm with 3D Gaussian filtering for noise reduction. Semi-automatic 3D regions of interest (ROIs) were drawn using ITK-snap 2.2.0 and analysed using in-house software designed with MATLAB 2013a ((Mathworks, Natick, MA, USA).

To quantify the spatial distribution of pulmonary perfusion, voxels within the ROI for each slice following the dorsoventral, caudocranial and mediolateral axes were extracted to measure the number of voxels and total activity profile along each of those planes. The right and left lungs were included into one ROI for each axis. For the mediolateral axis, the right lung was flipped and translated to align geometrically with the left lung and included into one ROI.

Spatial distribution of uptake was determined by first computing the relative concentration for each slice as a function of cumulative voxels in the three different axes.

$$ \mathrm{Relative}\ \mathrm{Concentration}=\frac{\mathrm{Mean}\ \mathrm{activity}\ \mathrm{per}\ \mathrm{voxel}\ \mathrm{in}\ \mathrm{s}\mathrm{lice}}{\mathrm{Mean}\ \mathrm{activity}\ \mathrm{of}\ \mathrm{all}\ \mathrm{s}\mathrm{lice}\mathrm{s}\ \mathrm{in}\ \mathrm{R}\mathrm{O}\mathrm{I}}\times 100 $$

The difference between each slice relative concentration and the overall mean ROI activity (100 %) was then plotted as a function of cumulative voxels along each respective axis and reported as the relative concentration difference. Consequently, a slice with mean activity per voxel equal to mean activity of the ROI would have a difference of 0 %. A positive or negative difference, respectively, represents the mean percent higher or lower activity for each slice compared to the whole ROI mean slice activity.

To determine the activity gradient along each axis, two distinct same-size compartments each containing 50 % of the voxels were created. The mean relative concentrations of the two compartments were subtracted to determine the gradient along each axis. Thus, equal activities in both compartments would give a gradient of 0. A positive or negative difference represents the respective percentage of decreasing of increasing gradient along the studied axis.

Additionally, to have a complementary graphical representation of the activity distribution along each axis, a graph of cumulative activity as a function of cumulative voxels was created. Quartiles of activity and their corresponding lung volumes at 25 % (VAQ25), 50 % (VAQ50) and 75 % (VAQ75) were determined as well as the area under the curve (AUC) of these graphics. Therefore, in the event where 25, 50 and 75 % of total activity is reached before or after 25, 50 and 75 % of total voxels or in the event where AUC is different from 5000 (AUC of a directly proportional situation between activity and volume), this would represent a non-uniform distribution with a perfusion gradient.

Finally, a semi-quantitative analysis was made to determine the distribution of activity intensity independently of the spatial distribution. To do so, a frequency histogram of activity intensity per voxel throughout the ROI was obtained. The voxels were grouped depending on their intensity in 15 consecutive bins of increasing 6.67 % activity steps from 0 % (no activity) to 100 % (maximum pixel activity in the ROI). A graph representing the frequency of voxels (equivalent to the percentage of lung volume) was then plotted as a function of the 15 intensity bins.

### Spatial distribution of ^99m^Tc-PulmoBind in a quadruped mammal

Five adult mongrel dogs weighing 9–13 kg were used in this study. Study protocol was approved by the animal research and ethics review board and conducted in accordance with the regulations and ethical guidelines from the Canadian Council for the Care of Laboratory Animals. After administration of 30 mg/kg pentobarbital sodium, the animals were intubated with an endotracheal tube. Anaesthesia was maintained with 1–3 % isoflurane. The animals were installed in supine position, and an IV catheter installed in a posterior paw vein for PulmoBind administration. The tracer was injected and a pulmonary SPECT and image analysis were performed as described above.

### Statistics analysis

Values are means and standard error of the mean (SEM) unless indicated otherwise. Differences from the PulmoBind-human group were evaluated by two-tailed *t* tests. The pulmonary activity-time curve of ^99m^Tc-PulmoBind was integrated from appearance to peak. Peak activity and the integrated activity were corrected for body surface area. Both corrected and uncorrected parameters were correlated with age, height, weight, plasma creatinine and respiratory function test parameters using a Pearson correlation matrix. Analysis was performed using SAS 9.3 and GraphPad Prism 6 software.

## Results

**Fig. 1:**
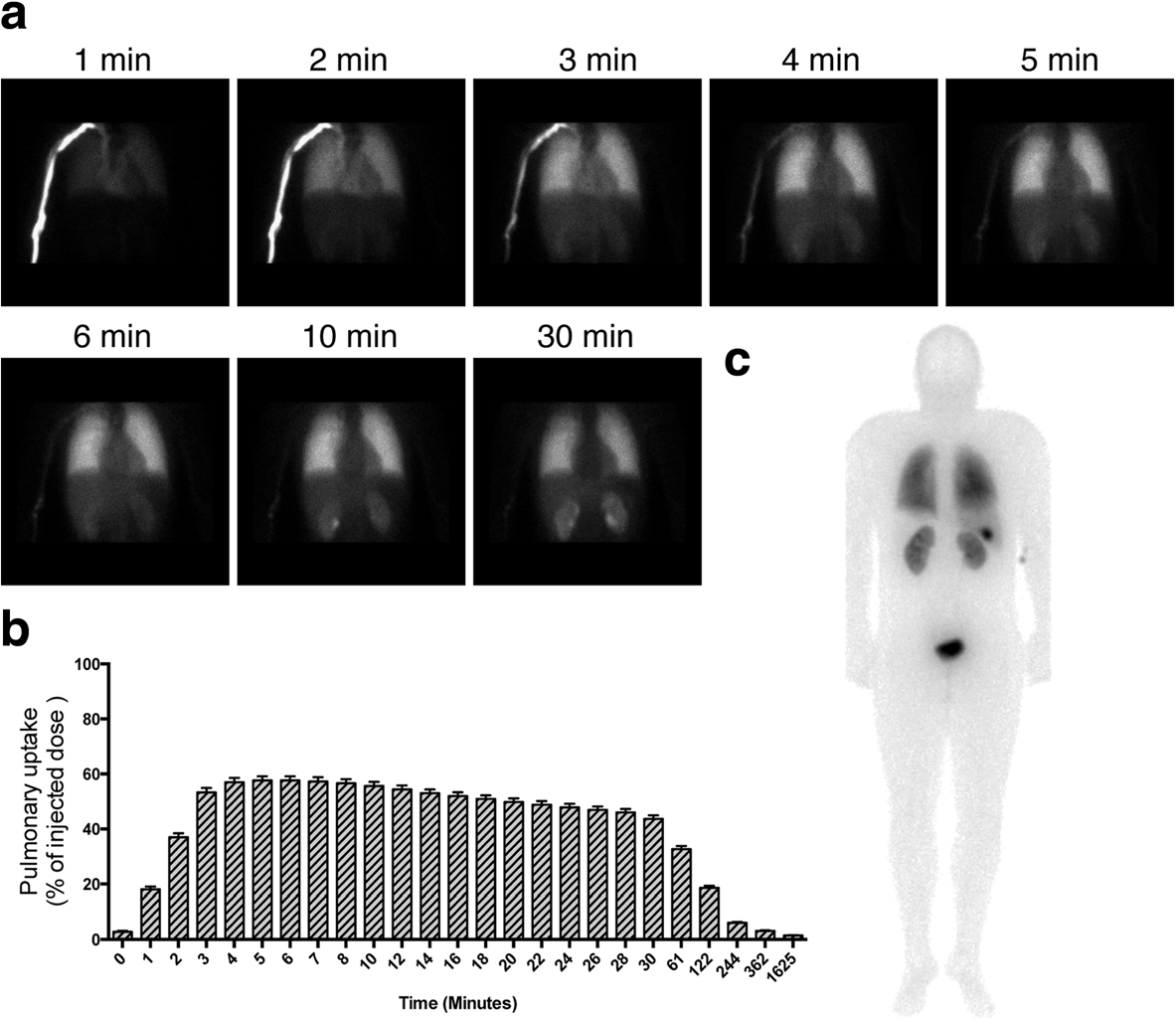
Dynamic SPECT imaging of pulmonary perfusion with ^99m^Tc-PulmoBind in human. **a** Dynamic SPECT imaging after forearm vein injection of ^99m^Tc-PulmoBind in a human subject. **b** Lung biodistribution of ^99m^Tc-PulmoBind at various times in human subjects (*n* = 19). **c** Whole body planar scintigraphy in a human 30 min after injection (posterior view)

As previously reported, labelled PulmoBind was prominently retained by the lungs with a peak uptake of 58 % ± 1 % of the injected dose occurring 5.5 ± 0.2 min following injection with an area under the activity-time curve (AUC) of 9089 ± 344. Kinetic analysis (Fig. 1) reveals rapid lung uptake with very slow decay of activity that remains at 44 % ± 1 % after 30 min and 33 % ± 1 % after 60 min. Whole body planar imaging at 60 min demonstrates persistent lung activity with elimination of the tracer by the kidneys and the liver as evidenced by urinary bladder and gallbladder activities. Complete safety and dosimetric analysis of ^99m^Tc-PulmoBind were previously reported [13].

In a new correlation analysis, we find that Peak ^99m^Tc-PulmoBind uptake by the lungs and the area under the activity-time curves were inversely correlated with age and positively correlated with respiratory function parameters (Table 1). In addition, the integrated activity, but not peak uptake, correlated with body weight and height and with plasma creatinine. When parameters were indexed for body surface area, only age remained weakly inversely correlated with both indices, all other correlations becoming non-significant. This unique correlation was lost when the only two female subjects, which were also the oldest subjects, were removed from the analysis.

**Table 1:** Correlation of Peak ^99m^Tc-PulmoBind uptake and of integrated pulmonary activity-time curves with physiological and respiratory function parameters

	Correlation coefficients			
	Peak uptake	Integrated uptake	Indexed peak uptake	Indexed integrated uptake
Age	−0.76***	−0.59*	−0.49*	−0.48*
Creatinine	0.38	0.49*	0.19	0.45
Weight	0.41	0.50*	−0.34	0.42
Height	0.44	0.50*	−0.16	0.45
FVC	0.53*	0.61**	0.002	0.35
FEV_1_	0.60**	0.69***	0.01	0.39
FEV_1_/FVC	0.51*	0.55*	0.02	0.28
PEF	0.48*	0.59**	−0.24	0.18

Parameters in the two right columns were indexed to body surface area

*FC* forced vital capacity; *FEV*_*1*_ forced expiratory volume in 1 s; *PEF* peak expiratory flow

*p < .05, **p < .01, ***p < .001

### Spatial distribution of PulmoBind as a function of cumulative voxels in the different planes

**Fig. 2:**
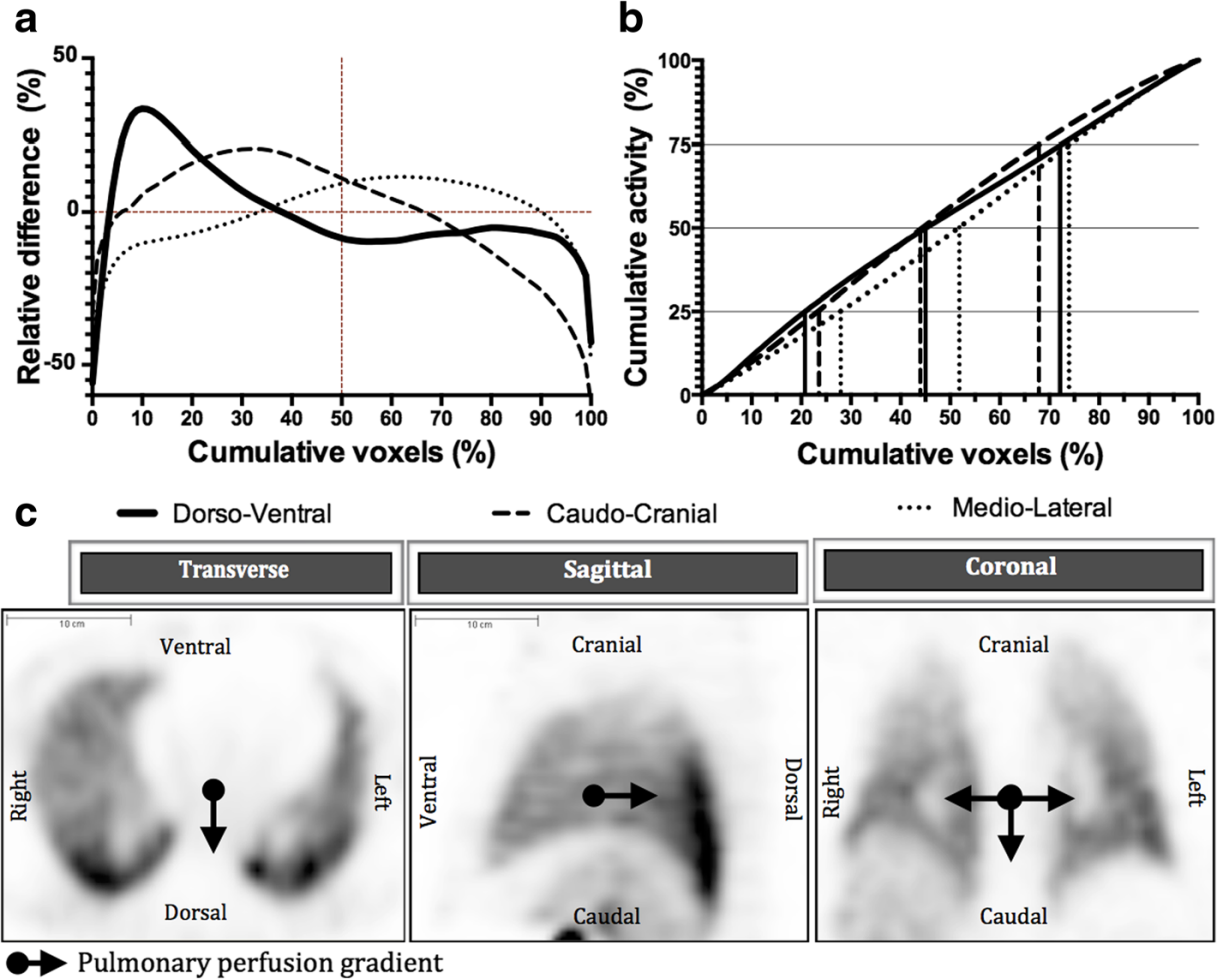
Pulmonary spatial distribution of ^99m^Tc-PulmoBind in healthy humans (*n* = 19). **a** Comparison of the relative concentration difference as a function of cumulative voxels in dorsoventral, caudocranial and mediolateral axes. **b** Cumulative relative activity as a function of cumulative voxels the three planes. **c** Representative SPECT imaging of spatial distribution in transverse, sagittal and coronal planes in subject 006. The *arrows* point in the direction of perfusion gradients

**Fig. 3:**
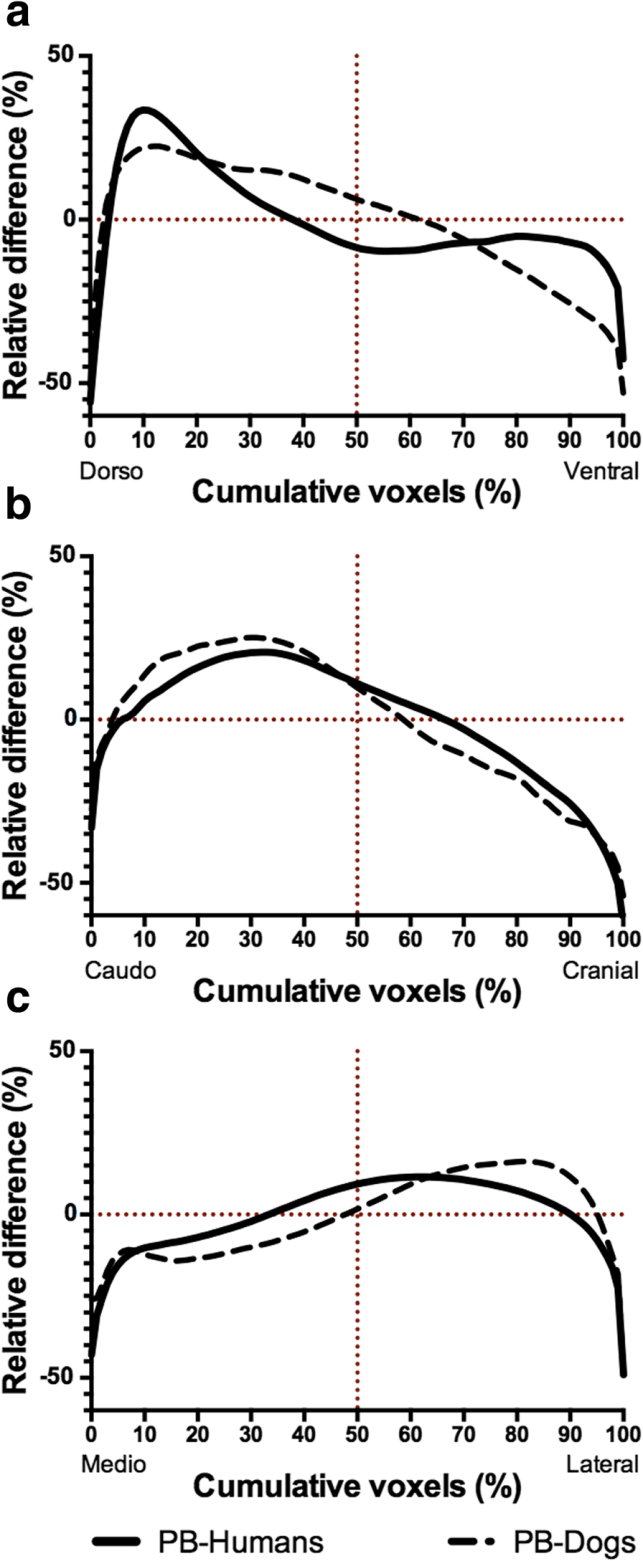
Comparative distribution of lung perfusion in humans and dogs. Comparison of the relative concentration difference as a function of cumulative voxels between PulmoBind-humans (*n* = 19) and PulmoBind-dogs (*n* = 5) in dorsoventral (**a**), caudocranial (**b**) and mediolateral axes (**c**)

The PulmoBind relative concentration difference and cumulative activity in the different planes are shown in Fig. 2 and Table 2. Overall, there was greater activity in the dorsal and caudal regions of the lungs compared to the ventral and cranial regions. There was almost even distribution in the mediolateral plane, the activity being slightly higher in the lateral regions. This creates a gradient of decreasing activity of 18.1 ± 2.1 % between the dorsal and ventral compartments and of 25.7 ± 1.6 % between the caudal and cranial compartments. Between the medial and lateral compartments, there was a mild positive gradient of 7.0 ± 1.0 %. Interestingly, the use of PulmoBind in supine dogs resulted in perfusion gradients similar to those observed in supine humans. The magnitude of the gradient was, however, greater in the caudocranial axis, followed by the dorsoventral and sagittal axis (Fig. 3 and Table 2).

**Table 2:** Comparison of pulmonary perfusion gradients in humans and dogs

	Dorsoventral (%)	Caudocranial (%)	Mediolateral (%)
PulmoBind-humans	18.1 ± 2.1	25.7 ± 1.6	−7.0 ± 1.0
PulmoBind-dogs	28.6 ± 2.5*	34.1 ± 5.0*	−18.1 ± 2.0**

*p < .05, **p < .001 vs. PulmoBind-humans

### Cumulative PulmoBind concentrations as a function of cumulative voxels in the different planes

**Fig. 4:**
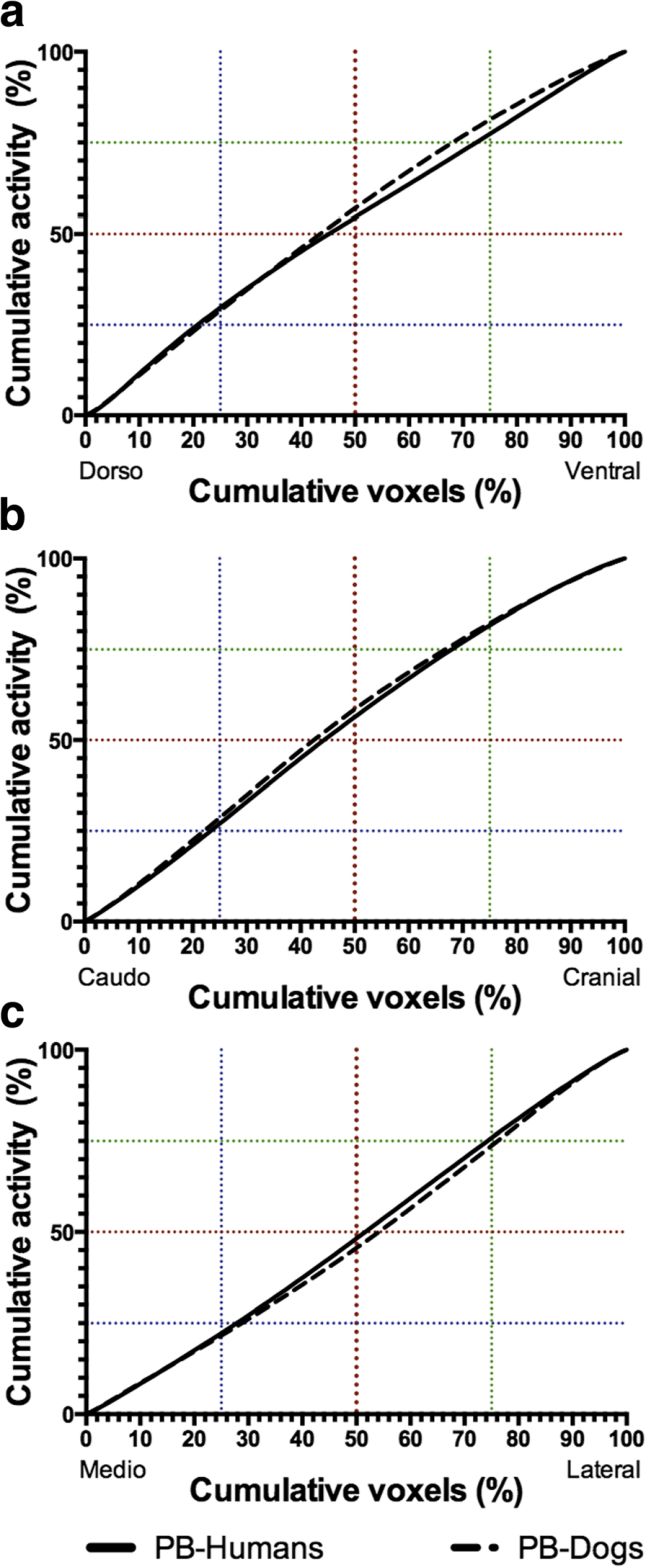
Cumulative perfusion activity as a function of lung volume in humans and dogs. Cumulative relative activity as a function of cumulative voxels in dorsoventral (**a**), caudocranial (**b**) and mediolateral (**c**) axes for PulmoBind in humans (*n* = 19) and PulmoBind in dogs (*n* = 5)

Cumulative quartiles representing 25, 50 and 75 % of total lung activity and their respective corresponding cumulative % voxels and AUCs are presented in Table 3 and Fig. 4 for the three axes. For PulmoBind in humans, there is asymmetrical distribution of activity as each activity quartile is reached before its respective cumulative voxels quartile in the dorsoventral and the caudocranial axis. There was again more even distribution in the mediolateral plane, as quartiles of activity were only slightly higher than corresponding cumulative voxels. Results for PulmoBind in dogs also showed evident gradients of a greater magnitude than those in humans.

**Table 3:** Volume of activity quartiles and areas under the cumulative activity curves

		Dorsoventral	Caudocranial	Mediolateral
VAQ25 (%)	PulmoBind-humans	20.9 ± 0.4	23.3 ± 0.2	27.8 ± 0.2
	PulmoBind-dogs	21.6 ± 0.5	22.1 ± 1.1	28.8 ± 0.5*
VAQ50 (%)	PulmoBind-humans	45.2 ± 0.6	44.4 ± 0.3	51.6 ± 0.2
	PulmoBind-dogs	43.5 ± 0.6	42.4 ± 1.1*	54.1 ± 0.5**
VAQ75 (%)	PulmoBind-humans	72.4 ± 0.4	67.9 ± 0.4	74.2 ± 0.2
	PulmoBind-dogs	68.0 ± 0.4***	66.6 ± 0.9	76.1 ± 0.2**
AUC-5000 (%^2^)	PulmoBind-humans	302 ± 34	395 ± 25	−92.5 ± 15.8
	PulmoBind-dogs	452 ± 39*	502 ± 80	−223 ± 21**

Cumulative voxels representing 25 % (VAQ25), 50 % (VAQ50) and 75 % (VAQ75) of cumulative ^99m^Tc-PulmoBind activity for each axis. AUC-5000 represents the deviation from a line of identity indicative of a positive or negative gradient vs. PulmoBind-humans

*p < .05, **p < .01, ***p < .001 vs. PulmoBind-humans

### Frequency histograms of lung activity for PulmoBind

**Fig. 5:**
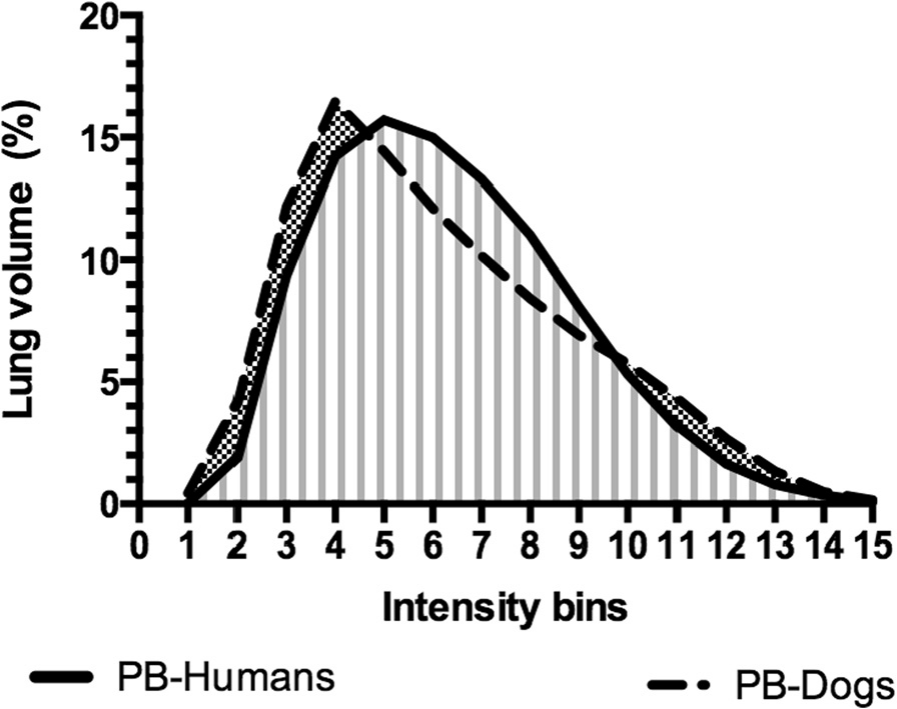
Frequency distribution of ^99m^Tc-PulmoBind in the human and canine lungs. Percentage of lung volume as a function of increasing activity bins in the lungs as obtained for PulmoBind in humans (*n* = 19) and PulmoBind in dogs (*n* = 5). Bins are numbered 1 to 15 at every 6.67 % cut-off level of the maximal pixel radioactivity from 0 % (no activity) to 100 % (maximum)

The percentage of lung volume for each of 15 increasing intensity bins for PulmoBind in humans and dogs are shown in Fig. 5. The frequency distribution of PulmoBind intensity in humans and dogs was similar, displaying a standard bell curve with unimodal distribution slightly skewed to the left lower intensity bins.

## Discussion

The spatial distribution of pulmonary blood flow is spatially heterogeneous owing to both gravity-dependent and gravity-independent factors. In human subjects, physical tracers such as radiolabelled MAA with SPECT imaging or intravascular contrast agents with computed tomography or magnetic resonance imaging have been used to study the distribution of blood flow. The aim of the current study was to use, for the first time, a metabolic tracer of pulmonary perfusion to determine the spatial distribution of lung microcirculatory perfusion. To explore the effects of gravity and species-specific lung geometry, we compared to the results obtained in a quadruped mammal (dogs) also in the supine position. We found that the spatial distribution of PulmoBind was heterogeneous with a predominant uptake in the more gravity-dependent dorsal and caudal regions of supine humans. There was consequently pronounced dorsoventral and caudocranial gradients of perfusion. There was also a milder mediolateral gradient. In supine dogs, the distribution of PulmoBind showed similar gradients, but of greater magnitude. Our findings therefore confirm the presence of a postural perfusion gradient, in the direction of gravity, present in both humans and in quadrupeds lying supine.

PulmoBind is derived from human adrenomedullin and specifically binds to the adrenomedullin receptor. This receptor, a heterodimer composed of the calcitonin-like receptor and the receptor activity modifying protein 2 (CLR-RAMP2), is densely distributed in the alveolar capillaries of the human lungs. Accordingly, adrenomedullin is rapidly extracted upon its first pass through the pulmonary circulation. PulmoBind uptake by the lung is therefore modified not only by blockage of the large pulmonary arteries, but can also detect blockage of small pulmonary arterioles in models of pulmonary arterial hypertension such as the monocrotaline model [14] and the hypoxia-sugen model [15]. As such, PulmoBind is a metabolic tracer and its distribution is related of lung microcirculatory perfusion and endothelium integrity. Consequently, a potential pitfall to the use of PulmoBind for the evaluation of perfusion would be the lack of uptake due to adrenomedullin receptor downregulation or desensitization, rather than reduced perfusion of capillaries. This differs from MAA which distribution relies exclusively on their physical properties. Approximately 500,000 MAA particles varying in size from 10 to 90 μm are intravenously injected and get trapped into the microcirculation following the distribution of pulmonary blood flow, which will temporarily occlude 1 in 1000 pulmonary arterioles. The number of particles injected and their size will vary between tests. Radiolabelled MAA have been used to semi-quantitatively assess the spatial pulmonary perfusion in normal lungs [1, 16–19]. Similar to these previous studies, but using a different methodological approach, we observe gravity-dependent and gravity-independent distribution of MAA in humans. Previous investigators have hypothesized that isogravitational heterogeneity resulted from varying regional vascular conductance at branching points [2, 20]. Indeed, studies performed in microgravity environment revealed that some lung perfusion heterogeneity persisted [21]. A novel fractal model incorporating isogravitational heterogeneity was proposed by Glenny [22–24] and suggests that as spatial resolution of the instruments of measure is improved, isogravitational perfusion heterogeneity is revealed [3]. Interestingly, PulmoBind activity gradients in dogs studied in the supine position were quite similar to those in humans but of higher magnitude, possibly due to anatomical differences in lung geometry and posture between species. The frequency distribution of PulmoBind lung activity (Fig. 5) showed a standard bell curve profile with unimodal distribution both in humans and in dogs. This suggests normal distribution of the adrenomedullin receptor in the pulmonary circulation of both species.

In a recent phase I study of PulmoBind, qualitative analysis of lung scans by experienced nuclear specialist revealed that PulmoBind imaging quality was superior to that historically obtained with MAA. Mean PulmoBind uptake by the human lungs showed little variability. After indexing for body surface area, uptake did not correlate with respiratory function parameters. The only significant correlation, although mild, was an inverse relationship with age. We also cannot exclude a relationship with gender as the only two female participants were the two older subjects driving this correlation. Future studies in a larger number of subjects will be necessary to explore this issue.

We used a novel approach to the evaluation of spatial distribution of SPECT imaging activity by computing relative concentrations and cumulative activity as a function of cumulative voxels in different axes. By using cumulative voxels, we reduced inter-individual variability and we show that this correlated well with distance along the lungs. Inherent limitations to the use of SPECT imaging are its lower resolution compared to MRI or CT and the occurrence of partial volume and edge effects.

Our findings could have clinical applications as the spatial distribution of lung perfusion is modified in physiologic and pathologic conditions. Using the multiple indicator-dilution technique in exercising dogs, we demonstrated that the metabolically active pulmonary vascular surface-area increased almost linearly with tripling of blood flow [12]. There is therefore a pulmonary vascular “reserve” that can accommodate the increase in cardiac output and expand the surface for gas exchange and metabolic functions. Lung vascular recruitment in response to increasing blood flow will accordingly modify the spatial distribution of pulmonary distribution with a reduction in the gravitational circulation component. Furthermore, evaluation of the distribution of pulmonary perfusion could be of value in the evaluation of lung disorders, such as pulmonary arterial hypertension. A recent study evaluating the distribution of MAA demonstrated that its distribution was modified in subjects with pulmonary arterial hypertension [25]. A phase II study of PulmoBind in subjects with pulmonary arterial hypertension will be evaluating this concept (Clinicaltrials.gov NCT02216279). A study of the spatial distribution of the metabolically active pulmonary circulation at rest and with increasing pulmonary blood flow, such as exercise, could provide a unique insight into the capacity of the lung to recruit vascular surface area. Conversely, variations in the spatial distribution of pulmonary perfusion in conditions associated with a loss of recruitable pulmonary perfusion, such as pulmonary arterial hypertension, could provide a unique method to diagnose and evaluate lung vascular dysfunction. It is estimated that more than 50 % of the pulmonary vascular bed is lost before pulmonary hypertension becomes hemodynamically detectable. Detections of variations in spatial distribution of flow may provide earlier detection of the disease process.

## Conclusions

We used an adrenomedullin receptor ligand to evaluate the spatial distribution of the perfused lung microcirculation in the supine human. The perfusion was heterogeneous with a predominant distribution in the more gravity-dependant dorsal and caudal lung regions. A similar gradient was confirmed in supine dogs. Future studies are needed to determine if this novel endothelial cell tracer could be used to detect physiologic and pathologic variations of lung perfusion in conditions such as pulmonary hypertension.

## Abbreviations

^99m^Tc, technetium-99m; AUC, area under the curve; CT, computed tomography; MAA, macroaggregates of albumin; MRI, magnetic resonance imaging; ROI, region of interest; SPECT, single-photon emission computed tomography; VAQ, volume of activity quartiles.
